# Quantitative Evaluation of Carotid Artery Stenosis by Multi‐VENC 4D Flow MRI: Incorporating Turbulent Kinetic Energy for Clinical Validity

**DOI:** 10.1002/jmri.70008

**Published:** 2025-06-29

**Authors:** Takahiro Ando, Tetsuro Sekine, Satoshi Suda, Kentaro Suzuki, Yasuo Murai, Kotomi Iwata, Masatoki Nakaza, Masashi Ogawa, Makoto Obara, Gerard Crelier, Kazumi Kimura, Shin‐ichiro Kumita

**Affiliations:** ^1^ Department of Radiology Nippon Medical School Tokyo Japan; ^2^ Department of Neurology Nippon Medical School Tokyo Japan; ^3^ Department of Neurological Surgery Nippon Medical School Tokyo Japan; ^4^ Philips Electronics Japan Healthcare Tokyo Japan; ^5^ GyroTools LLC Zurich Switzerland

**Keywords:** 4D flow MRI, blood flow volume, carotid artery stenosis, plaque characterization, turbulent kinetic energy

## Abstract

**Background:**

Cervical internal carotid artery stenosis (ICS) is a recognized risk factor for ischemic stroke, yet morphological severity alone may not fully reflect hemodynamic alterations. Turbulent kinetic energy (TKE), derived from multi‐velocity‐encoding (multi‐VENC) four‐dimensional (4D) flow MRI, may provide a robust marker for ICS assessment, though its utility in carotid arteries remains underexplored.

**Purpose:**

To investigate the reproducibility of TKE measurement and to assess correlations with MR angiography (MRA)‐derived stenosis, black blood T1‐weighted imaging (T1BB)‐derived plaque scale, and ultrasound parameters.

**Study Type:**

Prospective.

**Population:**

Twenty‐three patients (6 [26%] female; median age: 72 years, IQR: 60–80) with suspected ICS based on cerebrovascular symptoms or screening carotid ultrasound.

**Field Strength/Sequence:**

3‐T, multi‐VENC (33–100–300 cm/s) 4D flow MRI of the carotid arteries at 1.0 mm^3^ isotropic resolution, using k‐space–time principal component analysis (k–t PCA) acceleration, time of flight‐MRA (3D gradient‐echo), and T1BB (turbo spin echo).

**Assessment:**

Two neuroradiologists measured TKE once per case for interobserver evaluation. TKE was measured in a volume from just proximal to the bifurcation and slightly distal to the ICA stenosis. TKEbeat was defined as the total TKE integrated over the cardiac cycle. Stenosis and plaque features were assessed by MRA and T1BB, respectively. Carotid ultrasound parameters included peak systolic velocity, resistance index, intima‐media thickness (IMT), and plaque characteristics.

**Statistical Tests:**

Intraclass correlation coefficient (ICC) and Bland–Altman analyses were used for interobserver agreements. Associations between TKEbeat and conventional parameters were evaluated using Spearman's rank correlation. TKEbeat was compared between subgroups based on stenosis, plaque grade, and vascular risk factors using Mann–Whitney *U*‐tests. Significance threshold: *p* < 0.05.

**Results:**

The ICC was 0.922 for TKEbeat. TKE correlated with stenosis (*r* = 0.309), plaque scale (*r* = 0.392), and IMT (*r* = 0.543). TKEbeat was higher in the stenosis group.

**Data Conclusion:**

Multi‐VENC 4D flow MRI enables reproducible TKE measurement correlated with carotid stenosis and plaque features.

**Evidence Level:**

Level 1.

**Technical Efficacy:**

Stage 1.

**Summary**

- Plain language summary
  ○ This study examined how blood flow turbulence and volume change in patients with narrowing of the internal carotid artery, a key blood vessel supplying the brain.
  ○ Using an advanced MRI technique called 4D flow MRI, the researchers measured turbulent kinetic energy (TKE) and blood flow volume (BFV).
  ○ They found that these measurements were consistent between different observers and that TKE increased with more severe artery narrowing and plaque features, while BFV decreased.
  ○ These findings suggest that TKE and BFV may help doctors better understand blood flow changes and improve stroke risk assessment in patients with carotid artery disease.

## 1 Introduction

Cervical internal carotid artery stenosis (ICS) is a risk factor for ischemic stroke, accounting for 10%–20% of all strokes or transient ischemic attacks (TIAs) [1]. To stratify stroke risk in ICS, the morphological degree of stenosis has been widely used [2]. Previous studies have demonstrated correlations between the severity of morphological stenosis and stroke [3, 4]. In addition to the degree of stenosis, blood flow characteristics are associated with stroke risk [5]. At the stenotic vessel, morphological stenosis severity is not always linearly correlated with reductions in blood flow volume (BFV), which also influences stroke risk [6, 7]. Blood flow hemodynamics also affects both the formation and rupture of vulnerable plaques that independently increase stroke risk, even when stenosis is only mild to moderate [8, 9, 10, 11, 12].

Time‐resolved four‐dimensional phase‐contrast MRI (4D flow MRI) can measure blood flow velocity, flow volume, and wall shear stress (WSS), and it can also visualize blood flow path lines [13, 14, 15, 16, 17]. It is expected that 4D flow MRI can help predict outcomes in ICS by revealing hemodynamic alterations around stenotic lesions [18, 19]. However, this modality has inherent limitations, especially in stenotic areas, because measurement errors in flow velocity and other parameters (e.g., WSS) may occur due to relatively low spatial resolution [20].

Turbulent kinetic energy (TKE) represents the energy contained in the microscopic, chaotic velocity fluctuations that arise when blood flow becomes turbulent. In physical terms, it is the “budget” of kinetic energy stored in swirling eddies; clinically, it can be regarded as a surrogate for energy dissipation and flow complexity. In cardiac imaging, TKE measured with 4D flow MRI has been linked to irreversible pressure loss in aortic stenosis and abnormal ventricular workload in hypertrophic cardiomyopathy [21, 22]. Several other reports have also investigated TKE in the cardiac and great vessel regions [23, 24, 25, 26, 27, 28, 29, 30]. Carotid bifurcation flow is likewise prone to transition from laminar to turbulent, particularly downstream of a stenotic lesion, where elevated TKE may reflect hemodynamic stress that contributes to plaque destabilization and stroke risk. We therefore hypothesized that carotid TKE, together with BFV, would add complementary information to conventional imaging markers in patients with ICS. However, to date, no in vivo evaluation of TKE has been conducted in the carotid artery.

Against this background, this preliminary study aimed to investigate the interobserver measurement accuracy of TKE and BFV obtained from multi‐velocity‐encoding (multi‐VENC) 4D flow MRI, and to characterize these parameters in comparison with conventional clinical indicators measured by MRI and ultrasonography.

## 2 Materials and Methods

This prospective study conformed to the Declaration of Helsinki. The study protocol was approved by the ethics committee of Nippon Medical School Hospital, and written informed consent was obtained from all patients.

### 2.1 Patients

Twenty‐six patients who were clinically suspected of having ICS and underwent carotid MRI between December 2019 and August 2020 were enrolled. Of these, 15 were asymptomatic, undergoing MRI for further evaluation of carotid stenosis detected by screening or checkup ultrasound. Eight were symptomatic, including two with amaurosis fugax, two with TIA, and four with acute ischemic stroke. Patients with a history of atrial fibrillation or MRA‐confirmed common carotid artery (CCA) stenosis were excluded, though none met these criteria.

Their past medical history—including sex, age, and carotid ultrasound findings and comorbidities such as hypertension (HT), hyperlipidemia (HL), and diabetes mellitus (DM) was confirmed by a review of electronic medical records, based on documented history and prescribed medications. Ultrasound parameters included peak systolic velocity (PSV), end‐diastolic velocity (EDV), mean velocity (Vmean), pulsatility index (PI), resistance index (RI), maximum intima‐media thickness (IMT), maximum plaque protrusion height, plaque surface grade (smooth, rough, irregular, or ulcerated), plaque signal category (hypoechoic, isoechoic, hyperechoic, or calcified), and plaque homogeneity (homogeneous and heterogeneous). Carotid ultrasound data were included if obtained within 6 months before or after 4D flow MRI.

### 2.2 Imaging

All patients were examined on the same 3‐T MRI system (Achieva; Philips Healthcare, Best, the Netherlands). First, a standard clinical carotid protocol—comprising time‐of‐flight MR angiography (TOF‐MRA) and black‐blood T1‐weighted imaging (T1BB)—was performed in accordance with the recommendations of the ASNR Vessel‐Wall Imaging Study Group and the 2023 guidelines of the European Society for Vascular Surgery [31, 32]. Immediately thereafter, a research 10‐min 4D flow MRI acquisition of the carotid arteries was obtained without contrast. The 4D flow MRI was acquired in the axial plane to leverage inflow enhancement, using the following parameters: repetition time (TR)/echo time (TE) = 8.4/5.4 ms; multi‐VENC = 33–100–300 cm/s; 15–21 heart phases; voxel size = 1.0 mm^3^ isotropic; k‐space–time principal component analysis (k–t PCA) factor of 5–7; total scan time ~ 10 min. The number of reconstructed cardiac frames was adapted to each patient's heart rate and ECG‐gating quality: 19 of the 23 data sets contained 15 frames, three contained 12 frames, and one contained 10 frames.

After imaging, TKE was calculated from the magnitude images of multi‐VENC data using Bayesian estimation (CRECON v4.6, Gyrotools LLC, Zurich, Switzerland), requiring approximately 15 min of offline reconstruction time. The multi‐VENC approach extended the dynamic range of TKE calculations [33, 34, 35]. The TOF‐MRA settings were as follows: TR/TE = 25/3.45 ms, flip angle = 18°, SENSE factor = 2, five slabs, and a voxel size of 0.28 × 0.28 × 0.70 mm^3^. T1 black‐blood (T1BB) imaging was performed with TR/TE = 491/31 ms, echo train length = 32, flip angle = 75°, motion‐sensitized driven equilibrium = 1 cm/s, and fat suppression.

### 2.3 4D Flow MRI Analysis

**FIGURE 1:**
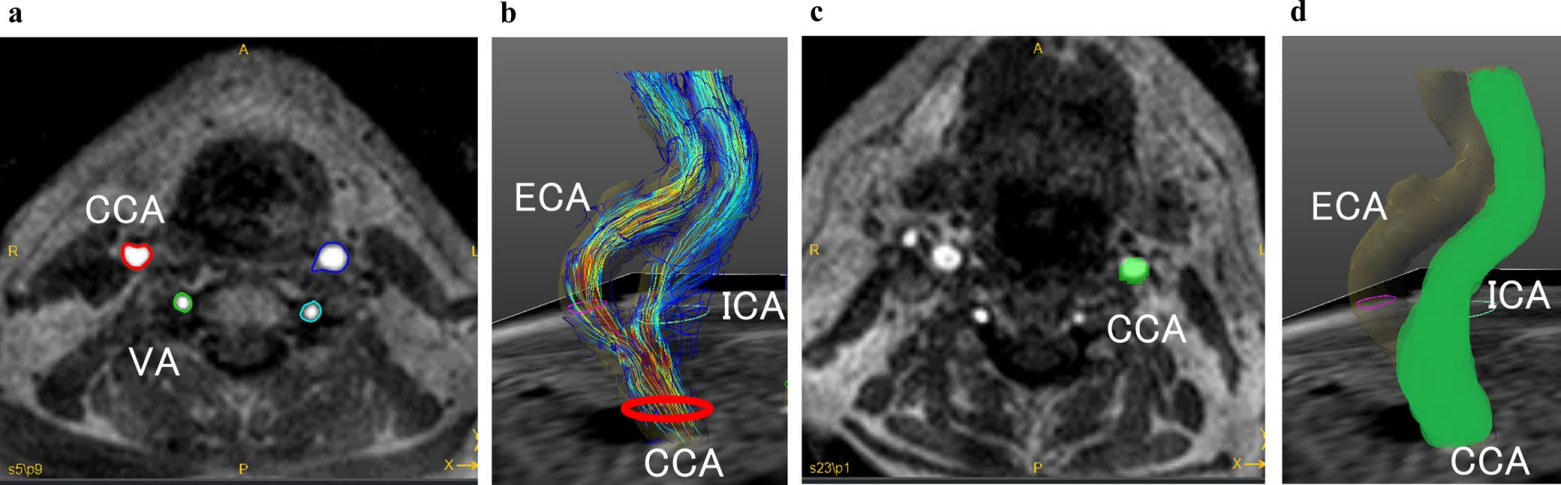
4D flow MRI magnitude images (a, c) and 3D images (b, d). BFV was measured by applying the semi‐automatic iso‐contour ROI in the proximal CCA (a, b). TKE was measured by manually defining the VOI from the CCA to the ICA (c, d). BFV = blood flow volume, CCA = common carotid artery, ICA = internal carotid artery, ROI = region of interest, TKE = turbulent kinetic energy, VOI = volume of interest.

Of the 26 patients, 13 (26 carotid arteries) were analyzed by two neuroradiologists (Takahiro Ando, 11 years and Tetsuro Sekine, 18 years) to evaluate interobserver variability, while the remaining cases were analyzed by a single neuroradiologist (Takahiro Ando). All data were processed using GT Flow software (version 3.1.0; GyroTools, Zurich, Switzerland). Two‐dimensional (2D) analysis planes were manually placed perpendicular to the vessel's long axis in the CCA and internal carotid artery (ICA) using magnitude images for anatomical guidance. BFV was measured in the distal CCA—chosen for its more uniform lumen geometry and stable flow signal—to ensure reliable segmentation and reproducibility across readers. Regions of interest (ROIs) were defined using a semi‐automatic iso‐contour method (Figure 1). The software automatically computed BFV (mL/s) throughout a cardiac cycle, and the mean BFV (average BFV over the cycle) was derived. For TKE measurements, a volume of interest (VOI) was manually drawn on the magnitude images, extending from the distal CCA just proximal to the bifurcation to the ICA slightly distal to the stenosis (Figure 1). TKEbeat was then calculated as the sum of voxel‐wise TKE values across a complete cardiac cycle; it represents the cumulative TKE dissipated during one heartbeat and is regarded as a surrogate marker of irreversible pressure loss and turbulence‐related WSS.

### 2.4 TOF‐MRA Analysis

All TOF‐MRA images were reviewed by a single neuroradiologist (Takahiro Ando, 11 years of experience). The degree of cervical ICA stenosis (%) was determined using TOF‐MRA maximum intensity projection (MIP) images based on the North American Symptomatic Carotid Endarterectomy Trial (NASCET) method [4]. Arteries were categorized into a stenosis group (≥ 50% stenosis, i.e., high‐grade or high‐moderate stenosis) or a non‐stenosis group (< 50% stenosis, i.e., low‐moderate, mild, or none).

### 2.5 T1BB Plaque Analysis

All black‐blood T1‐weighted images (T1BB) were reviewed by the same neuroradiologist. Using the slice that exhibited the greatest luminal narrowing, plaque signal intensity was graded on a 5‐point visual scale: Grade 1, markedly lower than the ipsilateral submandibular gland; Grade 2, slightly lower; Grade 3, equal; Grade 4, slightly higher; and Grade 5, markedly higher. This 5‐point grading scheme was adapted from multicontrast vessel‐wall MRI studies demonstrating that low T1BB signal corresponds to calcification or fibrous tissue, iso‐intensity to lipid‐rich necrotic core (LRNC), and high signal to intraplaque hemorrhage (IPH) [36, 37].

For quantitative assessment, the plaque‐to‐gland ratio (p/g ratio) was calculated by placing circular ROIs within the plaque and within the ipsilateral submandibular gland on the same slice, the latter being chosen for its stable T1 signal across subjects [36]. All evaluations and measurements, including MRA‐derived stenosis grading and T1BB‐based visual and quantitative assessments, were performed using WeVIEW (Fujifilm, Tokyo, Japan), a general‐purpose DICOM viewer.

### 2.6 Subgroup Classification

For comparative analysis, arteries were categorized into subgroups based on clinical and imaging parameters, including:

Stenosis severity: moderate (< 50%) vs. severe (≥ 50%) stenosis, as determined by MRA.

Plaque visual scale: low‐grade (< 4) vs. high‐grade (≥ 4), as assessed from T1‐weighted black‐blood images.

Comorbidities: presence or absence of HT, HL, and DM, determined from medical records and medication history.

### 2.7 Statistical Analysis

To confirm the statistical independence of bilateral carotid measurements, linear mixed models were applied for each parameter using subject ID as a random effect and side (left/right) as a fixed effect. No significant side‐to‐side differences were observed (*p* > 0.05), and both sides were therefore treated as independent samples in subsequent analyses. All statistical calculations were performed using SPSS (version 25; IBM Corp., Armonk, NY, USA). All numerical data are presented as medians with 25th and 75th percentiles. Categorical variables are shown as counts (%). Interobserver agreement for 4D flow MRI analyses was evaluated using the intraclass correlation coefficient (ICC) and Bland–Altman plots [38]. Spearman's rank correlation was used to assess the relationship between TKEbeat and other parameters (e.g., NASCET stenosis and IMT). To compare values between subgroups, the Mann–Whitney *U*‐test was used. A *p* value < 0.05 was considered statistically significant.

## 3 Results

A total of 23 patients (46 carotid arteries) were included in this study (after excluding three patients who could not undergo 4D flow MRI due to unstable heart rates). Of these 23 patients, 11 underwent carotid ultrasonography within 6 months before or after 4D flow MRI. The median time interval from carotid ultrasound to 4D flow MRI was −2 days (interquartile range [IQR]: −48 to 3 days). Among the 46 carotid arteries analyzed (23 patients), 16 arteries (35%) showed ≥ 50% stenosis on TOF‐MRA (high‐grade or high‐moderate), whereas the remaining 30 arteries (65%) exhibited < 50% stenosis (low‐moderate, mild, or none).

**FIGURE 2:**
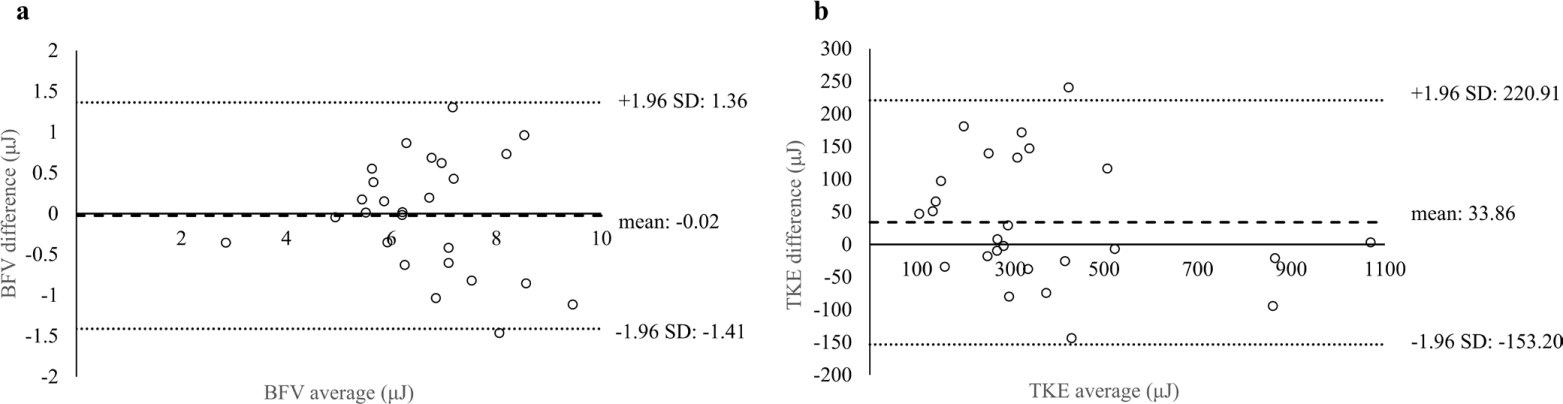
Bland–Altman plots of BFV (a) and TKEbeat (b). The thicker dashed line indicates the mean difference (bias), while the thinner dashed lines represent the 95% limits of agreement (±1.96 SD). Both BFV and TKEbeat demonstrated high interobserver agreement (BFV: Bias = −0.02, LOA = −1.41 to 1.36; TKEbeat: Bias = 33.86, LOA = −153.20 to 220.92). BFV = blood flow volume, LOA = limits of agreement, TKEbeat = an average of turbulent kinetic energy.

Table 1 presents patient demographics and imaging parameters. Interobserver reliability results are shown in Table 2 and Figure 2. In the Bland–Altman plots, BFV had a bias of −0.02 and limits of agreement (LoAs) of −1.41 to 1.36, while TKEbeat showed a bias of 33.86 and LoA of −153.20 to 220.91. The ICC [1, 2] was 0.875 (95% confidence interval [CI]: 0.740–0.942) for BFV and 0.922 (95% CI: 0.822–0.964) for TKEbeat, indicating high inter‐rater consistency for both parameters.

**TABLE 1:** Patient characteristics and flow parameter.

Age (years)	72 (60–80)
Sex	Male = 17 (74%), Female = 6 (26%)
Smoking	10 (43%)
Hypertension	14 (61%)
Diabetes	7 (30%)
Hypercholesterolemia	13 (57%)
Coronary artery disease	3 (13%)
Arterial fibrillation	1 (4%)
History of stroke	4 (17%)
History of TIA	0 (0%)
4D Flow MRI	
TKEbeat (μJ)	271.12 (210.02–360.76)
Mean BFV (mL/s)	6.47 (5.49–7.39)
TOF‐MRA	
Stenosis (%)	24 (0–50)
p/g ratio	0.98 (0.67–1.25)
Plaque visual scale (1: highly lower than gland, 2: mildly lower, 3: equal, 4: mildly higher, 5: highly higher)	1:4, 2:6, 3:6, 4:5, 5:4
Carotid ultrasonography	
PSV (cm/s)	63 (55–75)
EDV (cm/s)	14 (11–21)
Vmean (cm/s)	29 (26–36)
PI	1.70 (1.47–1.85)
RI	0.78 (0.72–0.81)
IMT (mm)	0.81 (0.69–0.99)
Size (mm)	2.97 (2.01–4.54)
Surface (1: smooth, 2: rough, 3: irregular, 4: ulcer)	1:10, 2:0, 3:14, 4:2
Brightness (1: low, 2: iso, 3: hyper, 4: calcification)	1:2, 2:11, 3:2, 4:13
Homogeneity (1: homogeneous, 2: heterogenous)	1:2, 2:26

Abbreviations: BFV = blood flow volume, EDV = end‐diastolic velocity, IMT = intima‐media thickness, Mean BFV = an average of BFV, PI = pulsatility index, PSV = peak systolic velocity, p/g ratio = ratio of plaque and gland, RI = resistance index, TKE = turbulent kinetic energy, TKEbeat = an average of TKE, Vmean = mean velocity.

**TABLE 2:** Inter‐observer agreement for the measurements in 4D flow MRI.

	ICC (2,1)		Bland and Altman	
	*r*	*p*	Bias	LoA
Mean BFV (mL/s)	0.875 (95% CI: 0.740–0.942)	< 0.001	−0.02	−1.41 to 1.36
TKEbeat (μJ)	0.766 (95% CI: 0.618–0.864)	< 0.001	33.86	−153.20 to 220.91

Abbreviations: BFV = blood flow volume, CI = confidence interval, ICC = intraclass correlation coefficient, LoA = limit of agreement, Mean BFV = an average of BFV, TKE = turbulent kinetic energy, TKEbeat = an average of TKE.

**FIGURE 3:**
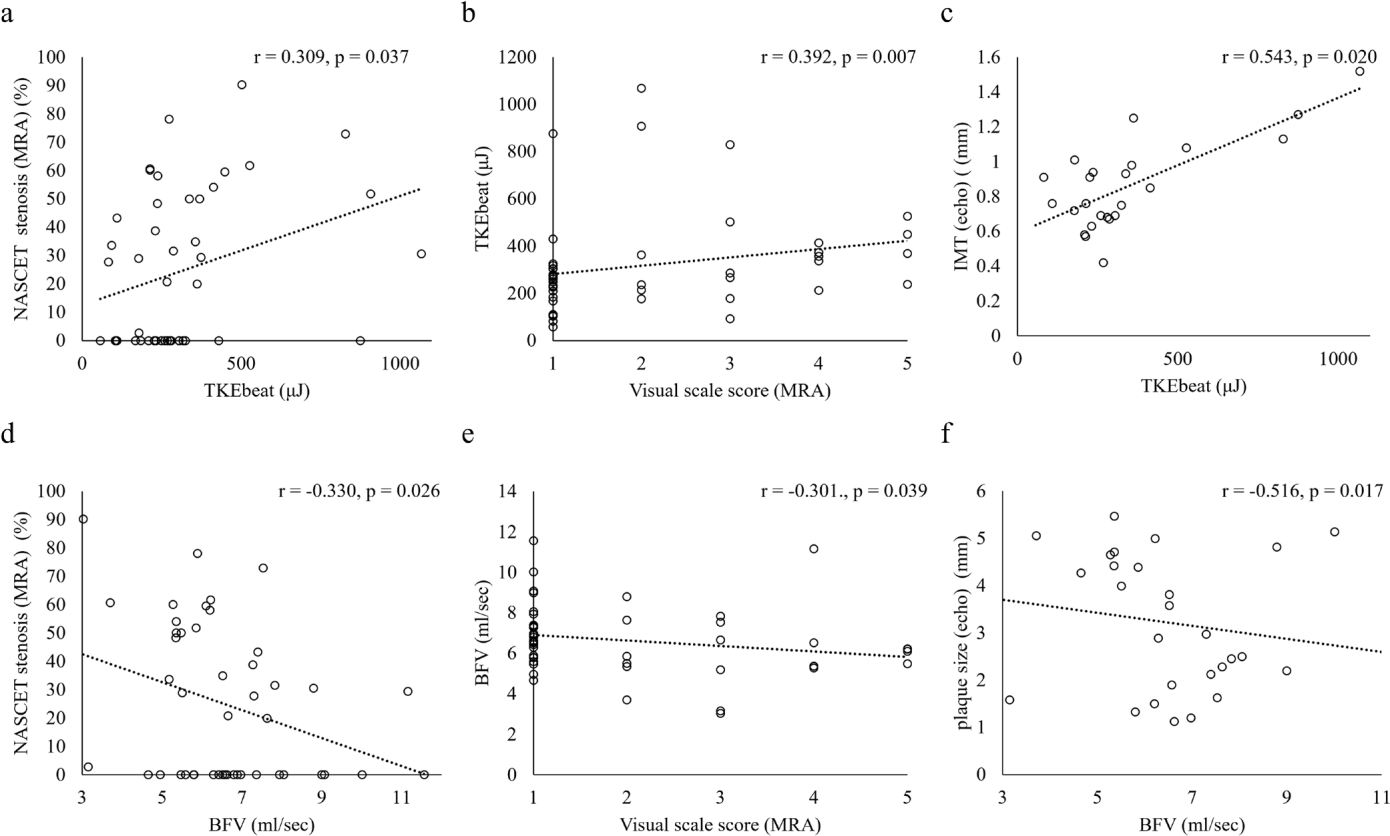
Correlation plots between TKEbeat or BFV and various parameters. TKEbeat showed significant correlations with stenosis (*r* = 0.309), plaque visual scale (*r* = 0.392), and IMT (*r* = 0.543). BFV demonstrated significant correlations with stenosis (*r* = −0.330), plaque visual scale (*r* = −0.301), and plaque size (*r* = −0.516). BFV = blood flow volume, IMT = intima‐media thickness, PSV = peak systolic velocity, r = correlation coefficient, TKEbeat = an average of turbulent kinetic energy.

Table 3 summarizes the correlations between BFV, TKEbeat, and various parameters from MRA and carotid ultrasonography. TKEbeat was significantly correlated with MRA‐derived stenosis (*r* = 0.309, 95% CI: 0.021–0.550), plaque visual scale (*r* = 0.392, 95% CI: 0.115–0.612), PSV (*r* = 0.462, 95% CI: 0.050–0.740), and IMT (*r* = 0.543, 95% CI: 0.157–0.785). Furthermore, BFV was significantly negatively correlated with MRA‐derived stenosis (*r* = −0.330, 95% CI: −0.566 to −0.044), plaque visual scale (*r* = −0.301, 95% CI: −0.544 to −0.012), and plaque size (*r* = −0.516, 95% CI: −0.770 to −0.121; Figure 3). No significant correlation was observed between TKEbeat and the p/g ratio (*r* = 0.286, *p* = 0.054), nor between mean BFV and the p/g ratio (*r* = −0.280, *p* = 0.060). Additionally, there were no statistically significant associations between TKEbeat and other plaque characteristics evaluated by ultrasound, such as surface irregularity, brightness, or homogeneity (*p* > 0.05 for all comparisons).

**TABLE 3:** Correlation with 4D flow parameter.

	TKEbeat		MeanBFV	
	*r*	*p*	*r*	*p*
MRI (*n* = 46)				
Stenosis	0.309 (95% CI: 0.021–0.550)	0.037	−0.330 (95% CI: −0.044 to −0.566)	0.026
p/g ratio	0.286 (95% CI: −0.005to 0.532)	0.054	−0.280 (95% CI: −0.527 to 0.011)	0.060
Plaque visual	0.392 (95% CI: 0.115–0.612)	0.007	−0.301 (95% CI: −0.544 to −0.012)	0.039
Carotid ultrasonography (*n* = 22)^a^				
PSV	0.462 (95% CI: 0.050–0.740)	0.031	0.299 (95% CI: −0.140 to 0.640)	0.178
EDV	0.177 (95% CI: −0.264 to 0.557)	0.430	0.069 (95% CI: −0.363 to 0.477)	0.759
Vmean	0.309 (95% CI: −0.129 to 0.646)	0.161	0.250 (95% CI: −0.192 to 0.608)	0.262
PI	0.166 (95% CI: −0.275to 0.549)	0.460	−0.060 (95% CI: −0.470 to 0.371)	0.791
RI	0.104 (95% CI: −0.332 to 0.504)	0.646	0.081 (95% CI: −0.353 to 0.486)	0.720
IMT	0.543 (95% CI: 0.157–0.785)	0.020	0.080 (95% CI: −0.354 to 0.485)	0.754
Size	0.242 (95% CI: −0.200 to 0.602)	0.290	−0.516 (95% CI: −0.770 to −0.121)	0.017
Surface	−0.158 (95% CI: −0.543 to 0.282)	0.493	−0.098 (95% CI: −0.499 to 0.338)	0.673
Luminance	0.249 (95% CI: −0.193 to 0.607)	0.263	−0.037 (95% CI: −0.452 to 0.391)	0.872
Homogeneity	−0.274 (95% CI: −0.624 to 0.167)	0.217	−0.249 (95% CI: −0.607 to 0.193)	0.263

Abbreviations: BFV = blood flow volume, CI = confidence interval, EDV = end‐diastolic velocity, IMT = intima‐media thickness, Mean BFV = an average of BFV, PI = pulsatility index, p/g ratio = ratio of plaque and gland, PSV = peak systolic velocity, RI = resistance index, TKE = turbulent kinetic energy, TKEbeat = an average of TKE, Vmean = mean velocity.

^a^ Carotid ultrasonography was performed within 6 months before and after 4D flow MRI.

**FIGURE 4:**
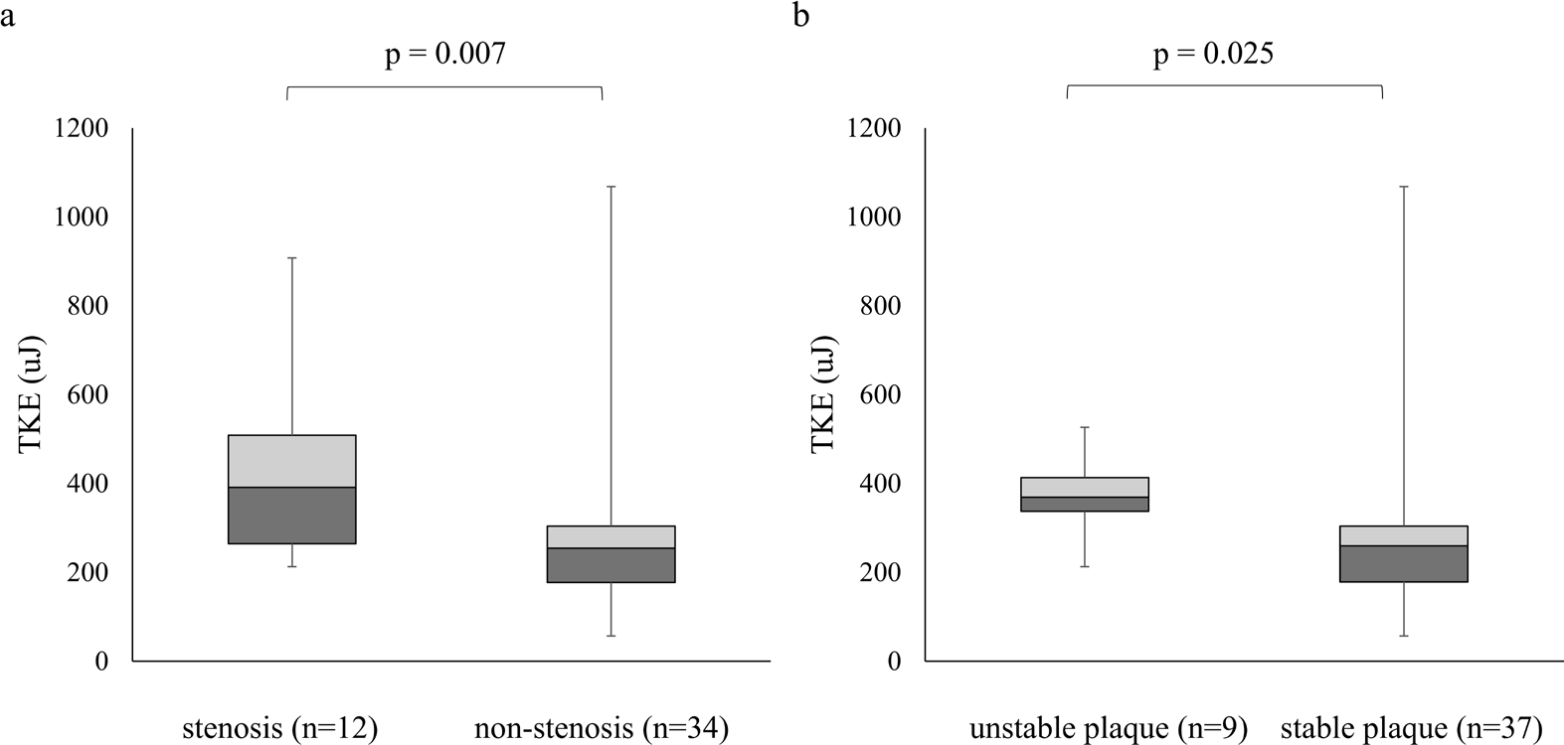
Box plots of TKEbeat categorized by stenosis degree and plaque visual scale. TKEbeat was significantly higher in the stenosis group (≥ 50%) than in the non‐stenosis group (< 50%), and also higher in the unstable plaque group (visual scale ≥ 4) than in the stable plaque group (visual scale < 4). MRA = magnetic resonance angiography, TKEbeat = an average of turbulent kinetic energy.

When arteries were divided into two groups based on stenosis degree (≥ 50% vs. < 50%) and the plaque visual scale (≥ 4 vs. < 4), TKEbeat showed significant differences. The stenosis group had a median TKEbeat of 391.50 μJ (IQR: 264.89–508.66 μJ), whereas the non‐stenosis group had 254.39 μJ (IQR: 177.49–304.48 μJ). Similarly, the unstable plaque group (≥ 4) had a median TKEbeat of 369.33 μJ (IQR: 337.43–413.66 μJ), while the stable plaque group (< 4) had 260.28 μJ (IQR: 178.64–304.53 μJ; Figure 4).

**FIGURE 5:**
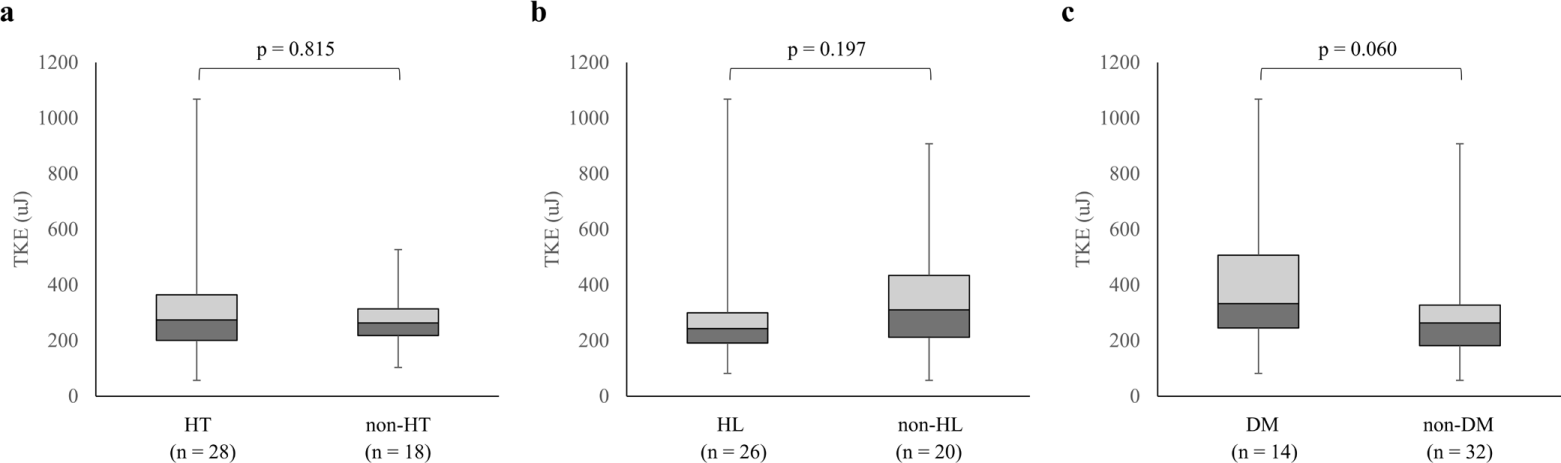
Box plots of TKEbeat based on the presence or absence of hypertension (HT), hyperlipidemia (HL), and diabetes mellitus (DM). No significant differences were observed in TKEbeat between groups with and without HT (*p* = 0.815), HL (*p* = 0.197), or DM (*p* = 0.060). TKEbeat = an average of turbulent kinetic energy.

**FIGURE 6:**
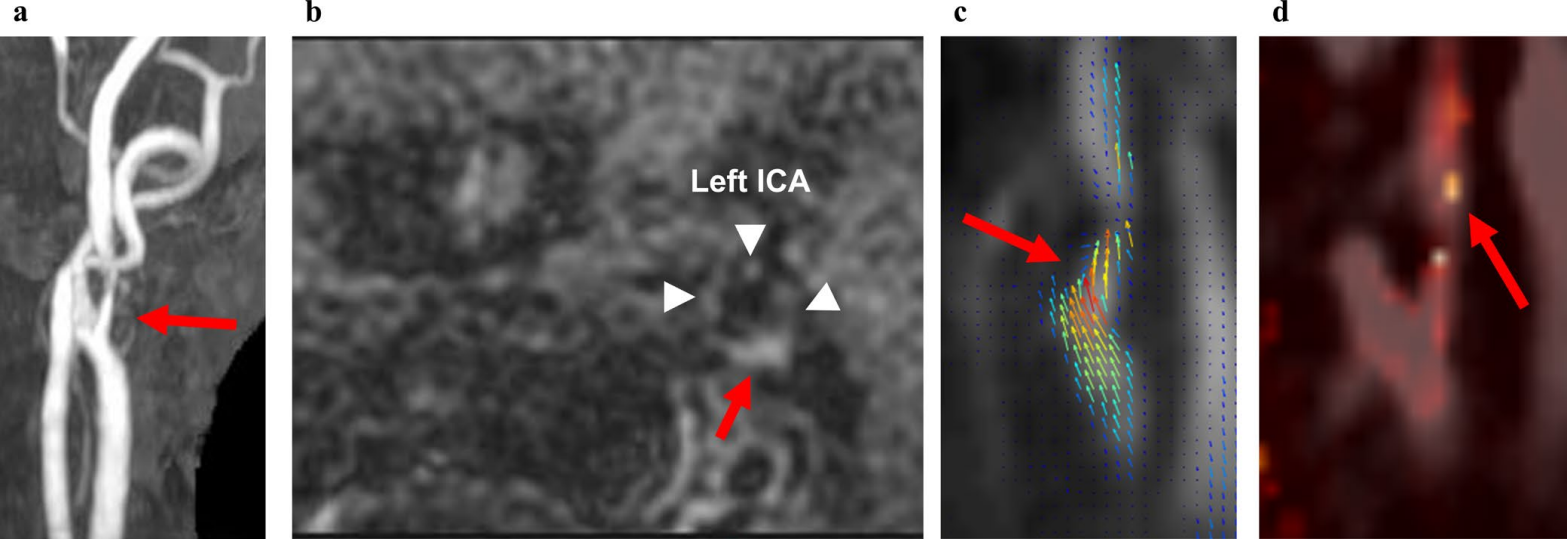
A representative case (78‐year‐old female with 72% left ICA stenosis). (a) TOF‐MRA MIP image; (b) T1BB image; (c) 4D flow MRI (vector overlay map); and (d) 4D flow MRI (TKE map). A 72% stenosis is observed at the origin of the left ICA (a, arrow). A high‐intensity plaque is identified at the corresponding site on T1BB (b, arrow; arrowheads indicate the left ICA). Jet flow is observed proximal and distal to the stenosis (c, arrow). TKE is markedly elevated distal to the stenosis, suggesting the presence of post‐stenotic turbulence (d, arrow). Although detailed assessment of local flow at the stenotic site is limited due to spatial resolution, the observed turbulence distal to the stenosis likely reflects disturbed flow at the stenotic segment. This finding may indicate a potential association between post‐stenotic turbulence and the development of unstable plaque. ICA = internal carotid artery, TKE = turbulent kinetic energy.

Figure 5 compares TKEbeat between groups with and without HT, HL, and DM, showing no significant differences (*p* = 0.815, 0.197, and 0.060, respectively). A representative case is shown in Figure 6.

## 4 Discussion

Our study shows that 4D flow MRI can quantify both TKEbeat and BFV in the cervical carotid arteries with excellent inter‐rater consistency. Beyond technical reliability, we found that TKEbeat increases in proportion to luminal narrowing and plaque signal intensity, while BFV decreases as stenosis and plaque burden progress. These complementary behaviors suggest that TKEbeat captures the turbulent component of hemodynamic stress at the lesion, whereas BFV reflects the global reduction in inflow—together providing a more comprehensive picture of stenosis‐related hemodynamics than morphological assessment alone.

In this study, we calculated the total TKE over a single cardiac cycle (i.e., TKEbeat). TKEbeat represents the total amount of turbulent energy dissipated during one heartbeat and serves as a surrogate marker of irreversible pressure loss and energy dissipation downstream of a stenosis [24, 25]. In 4D flow MRI, the number of acquired heart phases determines temporal resolution, such that a lower phase count may smooth out or miss turbulence peaks. Nonetheless, because TKEbeat is a time‐integrated metric covering the entire cardiac cycle, its sensitivity to modest variations in phase count is theoretically limited. By contrast, instantaneous metrics like peak TKE can be substantially underestimated if the true peak occurs between acquired frames. Indeed, some studies report underestimation of TKEbeat when heart phase count is reduced, but overall TKEbeat has been shown to be more robust than peak TKE against variations in imaging parameters [39, 40]. In our cohort, 19 of the 23 cases were acquired with 15 phases, 3 cases with 12 phases, and 1 case with 10 phases. Given that the majority of data sets used 15 phases, we believe this phase variation does not materially affect our TKEbeat comparisons, although further improvements in temporal resolution remain a future challenge.

In the setting of ICS, our findings on BFV are consistent with previous reports using ultrasound and 2D phase‐contrast MRI, which have shown a relationship between blood flow reduction and stenosis severity [6, 7]. Nonetheless, some studies suggested that peripheral flow and collateral circulation can be more important than the degree of stenosis itself for predicting stroke risk [7, 41]. Although our study focused on the anterior circulation, the principle of integrating stenosis evaluation with peripheral flow and collateral analyses—potentially using 4D flow MRI—could offer a more comprehensive risk assessment approach in clinical practice [17, 19, 41]. It has been reported that volume flow rates obtained with 4D flow MRI agree well with those measured by conventional 2D phase‐contrast MRI [42, 43]. In view of this existing validation, we did not acquire an additional 3D phase‐contrast data set in the present study; however, a larger follow‐up study is planned in which we will consider a direct head‐to‐head comparison.

Carotid artery turbulence has been investigated using particle image velocimetry, revealing that turbulence increases as stenosis worsens [44]. Hemodynamic factors such as WSS, turbulence, and local pressure gradients are believed to contribute to plaque formation and rupture [8, 9, 10, 11, 12]. Although TKE is derived from this fluctuation intensity, numerous studies have shown that elevated TKE can be used to estimate irreversible pressure loss and the turbulent component of WSS in vascular flows. In this study, TKE correlated not only with stenosis severity but also with vessel‐wall characteristics, indicating that it captures hemodynamic influences beyond those reflected by mean WSS alone. In line with this concept, Ziegler et al. demonstrated that near‐wall TKE obtained from 4D‐flow MRI shows a strong linear relationship to turbulent wall‐shear stress (tWSS)—the fluctuating component of WSS that is not represented by conventional mean‐WSS assessment [45]. Hence, elevated TKE may serve as an in vivo surrogate for the aggressive shear environment implicated in plaque destabilization. The lack of distinct associations between TKE and traditional risk factors such as HT, HL, and DM may further emphasize its potential as an independent measure of plaque‐related hemodynamics.

Although TKE measurements with 4D flow MRI have been more frequently studied in the heart and large vessels, our work represents an in vivo attempt to apply this technique to carotid artery stenosis [23, 24, 25, 26, 27, 28, 29, 30]. TKE is obtained from the reduction in magnitude‐signal intensity that occurs when microscopic velocity fluctuations induce intravoxel dephasing of the bipolar gradient—an effect formally analogous to diffusion‐weighted imaging [46, 47]. Because TKE is calculated from magnitude‐signal attenuation caused by intravoxel velocity fluctuations, it does not rely on phase information. Consequently, TKE is less sensitive to voxel size than phase‐derived metrics such as velocity, WSS, or viscous energy loss, making it particularly suitable for application in smaller vessels such as the carotid artery [40, 46, 48]. In this study, we used a multi‐VENC approach to extend the measurable range of both velocity and TKE while minimizing phase aliasing in the velocity data required for lumen segmentation [33, 34, 35, 48]. By combining a voxel size of 1.0 mm^3^ with the k–t PCA reconstruction method, we achieved clinically feasible scan times of approximately 10 min. The very high interobserver reproducibility of both TKE and BFV further supports the clinical utility of this approach.

4D flow‐derived TKE has been investigated in a wide spectrum of vascular beds—including left ventricular diastolic dysfunction, abdominal aortic stenosis, and Tetralogy of Fallot [23, 24, 25, 26, 27, 28, 29, 30]. These studies consistently demonstrate that TKE captures energy‐dissipating flow disturbances that elude conventional mean‐flow analysis. Our findings extend this paradigm to carotid artery disease, showing that high‐resolution, reproducible measurement of carotid TKE can provide complementary hemodynamic information to conventional stenosis grading.

The excellent interobserver agreement for both TKE and BFV highlights their reliability and repeatability in clinical settings. These features support the potential utility of 4D flow MRI‐derived markers as part of routine vascular evaluation. In particular, future studies that integrate TKE and BFV with demographic and clinical variables may enable more comprehensive hemodynamic assessment and improved risk stratification in patients with carotid artery disease. However, we recognize that the strength of the correlations between TKE and conventional imaging parameters is only moderate (*r* = 0.30–0.54), which limits their interpretive power. Accordingly, TKE should be regarded as a complementary rather than a standalone marker for assessing hemodynamic disturbances at present.

Finally, although the present protocol achieved sub‐millimeter isotropic resolution within a clinically practical scan time and yielded a very high inter‐rater agreement for both TKE and BFV, we recognize that agreement between readers alone does not guarantee absolute accuracy. Previous studies have reported good scan–rescan reproducibility for cerebral blood‐flow measurements at 0.9‐mm resolution and similarly good reproducibility for kinetic‐energy metrics in the cardiothoracic vasculature in vivo [49, 50]. In addition, measurements of TKE in a pulsatile stenosis phantom have shown excellent agreement with computational fluid dynamics simulations [40]. Because we did not perform a dedicated phantom experiment or an in vivo scan–rescan substudy, further validation will be essential to confirm measurement stability across scanners, platforms, and longitudinal time points.

### 4.1 Limitations

This study has several limitations.

First, the sample was small and skewed toward mild‐to‐moderate stenosis with relatively stable plaques, limiting generalizability; larger cohorts that include high‐grade and highly unstable lesions are required.

Second, BFV was quantified in the distal CCA rather than within the diseased ICA, so collateral pathways and external carotid runoff may have confounded the measurement. Although no CCA stenosis was detected on TOF‐MRA, subtle narrowing below the spatial resolution could still alter inflow conditions and slightly affect downstream TKE in the ICA. Future studies should explore direct ICA flow assessment using higher‐resolution 4D flow MRI protocols.

Third, cardiovascular risk factors such as smoking, HT, DM, and dyslipidemia were not included in the model, and unadjusted confounding may have weakened the associations between TKE/BFV and conventional metrics. Multivariable mixed‐effects modeling is warranted.

Fourth, plaque signal intensity on T1‐weighted black‐blood images was graded by a single reader; inter‐reader variability was therefore not assessed and may have introduced observer bias.

Fifth, the study relied on a single imaging session; scan–rescan and phantom validations are ongoing to establish the multi‐center and longitudinal stability of multi‐VENC 4D‐flow measurements.

Sixth, the analysis was cross‐sectional with few outcome events, so we could not assess whether TKE or BFV predicts future stroke/TIA. Large prospective longitudinal studies are needed to determine prognostic value.

Finally, our clinical protocol was limited to TOF‐MRA and T1‐weighted black‐blood imaging, chosen for their < 6‐min acquisition time and high sensitivity to IPH. The omission of T2‐weighted black‐blood and MPRAGE sequences may restrict detailed plaque characterization; incorporating a multi‐contrast protocol in future work could refine the assessment of plaque composition and its relationship to TKE/BFV.

## 5 Conclusion

Measurements of TKE and BFV using 4D flow MRI in patients with ICS may demonstrate high interobserver agreement and could show correlations with multiple established imaging findings.
